# Mid-Term Mortality Prediction Using Four Established Risk Scores in Patients with Chronic Limb-Threatening Ischemia Undergoing Cardiac Surgery

**DOI:** 10.3390/jcm14176210

**Published:** 2025-09-02

**Authors:** Yuki Setogawa, Shinsuke Kikuchi, Kyohei Oyama, Masahiro Tsutsui, Nobuyoshi Azuma, Hiroyuki Kamiya, Shingo Kunioka

**Affiliations:** 1 Department of Cardiac Surgery, Asahikawa Medical University, Midorigaoka Higashi 2-1-1-1, Asahikawa 078-8510, Japan; kyokui120086@gmail.com (Y.S.); koyama@asahikawa-med.ac.jp (K.O.); mt.0714.cuniculus@gmail.com (M.T.); 2 Department of Vascular Surgery, Asahikawa Medical University, Asahikawa 078-8510, Japan; kikuchi@asahikawa-med.ac.jp (S.K.); nazuma@asahikawa-med.ac.jp (N.A.)

**Keywords:** chronic limb-threatening ischemia, cardiac surgery, risk stratification, Japan SCORE, SPINACH SCORE, GNRI, frailty, mortality

## Abstract

**Objectives:** Patients with chronic limb-threatening ischemia (CLTI) represent a high-risk cohort for cardiac surgery due to the systemic atherosclerotic burden and frailty. This study aimed to evaluate the short- and mid-term prognoses of CLTI patients undergoing open cardiac surgery and to assess the prognostic utility of four risk scoring systems: Japan SCORE, SPINACH SCORE, Clinical Frailty Scale (CFS), and Geriatric Nutritional Risk Index (GNRI). **Methods:** We retrospectively analyzed 44 patients with CLTI who underwent open cardiac surgery between 2014 and 2023. Thirty-day and 1-year mortality were assessed. Patients were stratified using ROC-derived cutoffs for each scoring system. Kaplan–Meier survival curves and time-dependent ROC analyses were used to evaluate predictive performance over time. **Results:** Thirty-day mortality was significantly associated with a higher Japan SCORE; survivors had significantly lower scores than non-survivors (5.5% vs. 25.8%, *p* < 0.05). One-year mortality was significantly associated with nutritional status, as survivors showed a significantly higher GNRI than non-survivors (92.0 vs. 86.0, *p* < 0.05). Time-dependent ROC analysis revealed that the GNRI and SPINACH SCORE’s sustained prognostic accuracy beyond 1 year. Calibration plots showed good agreement between predicted and observed probabilities for the SPINACH SCORE and GNRI, while decision curve analysis (DCA) demonstrated that these two models provided greater net clinical benefit across a range of thresholds, particularly in the 5–20% range. **Conclusions:** Japan SCORE is effective for short-term risk prediction, while SPINACH SCORE and GNRI offer superior prognostic value for mid-term outcomes. These scoring systems may support preoperative risk stratification and decision-making in CLTI patients undergoing cardiac surgery.

## 1. Introduction

The number of patients with lower extremity arterial disease (LEAD) has been increasing in parallel with population aging. Chronic limb-threatening ischemia (CLTI), a clinical syndrome characterized by the presence of LEAD along with rest pain, gangrene, or a lower limb ulcer lasting more than two weeks, represents the most advanced stage of LEAD and is associated with high rates of mortality, limb amputation, and impaired quality of life [1]. The prognosis of patients with CLTI is generally poor, as evidenced by a 2-year survival rate of only 67.7% after disease onset [2]. It is therefore not surprising that patients with CLTI undergoing cardiac surgery may face even worse outcomes. This suggests that existing tools such as the SPINACH score—originally designed for patients undergoing isolated limb revascularization—may not fully capture the added complexity and mortality risk in this combined surgical population. In our previous report, the 2-year survival rate of such patients was only 43.9% [3].

Given the extremely high surgical risk in CLTI patients, careful patient selection is essential. In our previous study, we utilized the wound-ischemia and foot (WIfI) classification to stratify disease severity [4]. Although the WIfI classification is useful for assessing the severity of chronic limb-threatening ischemia, including wound, ischemia, and foot infection, it was primarily developed to predict amputation-free survival rather than overall mortality. Moreover, it is not routinely incorporated into cardiac surgical risk assessment tools, which may limit its practical application in this setting. In the present study, we evaluated four scoring systems to determine their ability to predict early and mid-term mortality in CLTI patients undergoing cardiac surgery: the Japan SCORE (for cardiac surgical mortality), the SPINACH SCORE (for mortality after revascularization in CLTI), the Clinical Frailty Scale (CFS), and the Geriatric Nutritional Risk Index (GNRI). The objective was to identify the most appropriate indicator for predicting postoperative prognosis in this high-risk population.

## 2. Methods

### 2.1. Ethical Approval

This study was approved by the ethics committees of our institute on 2 June 2025 (approval number: 19209) and performed in accordance with the guidelines laid down by the Declaration of Helsinki (1964). Given the retrospective, observational nature of the study design, the Ethics Committee waived the requirement of informed consent.

### 2.2. Study Design and Patient Selection

**Figure 1:**
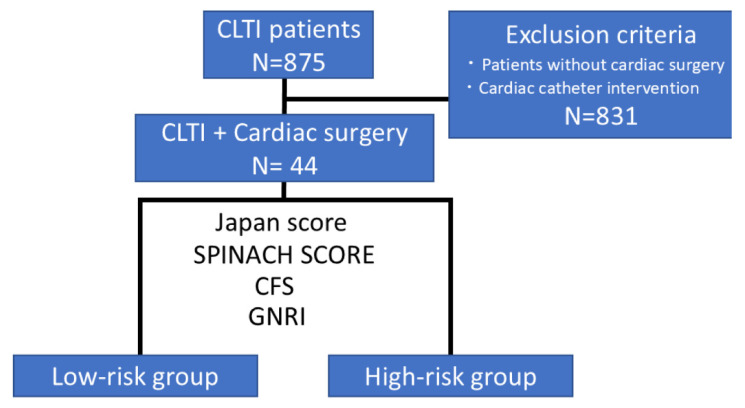
**Patient selection and study flow**. Of the 875 patients with CLTI, 44 patients were included in the present study after applying the exclusion criteria. Each patient was stratified into high- or low-risk groups using four scoring systems: the Japan SCORE, SPINACH SCORE, Clinical Frailty Scale (CFS), and Geriatric Nutritional Risk Index (GNRI).

Between April 2014 and March 2023, a total of 875 patients were hospitalized and treated for chronic limb-threatening ischemia (CLTI) at our institution. The diagnosis of CLTI was based on the documented diagnosis in electronic medical records. After excluding patients who did not undergo cardiac surgery and those who received catheter-based interventions such as transcatheter aortic valve implantation, 44 patients who underwent open cardiac surgery were included in this retrospective analysis (Figure 1). All types of open cardiac surgery were considered for inclusion. In this study, we investigated the short-term (30-day) and mid-term (1-year) mortality of these patients and evaluated the surgical outcomes of cardiac surgery in patients with CLTI by stratifying them into survivors and non-survivors. We further analyzed the prognostic factors influencing these outcomes. Outcomes, including 30-day and 1-year mortality, were determined based on documentation in institutional electronic medical records. All patients were actively followed at our institution throughout the study period, and no patients were lost to follow-up. Mortality status was directly extracted from physician notes and discharge summaries. No additional adjudication process was employed, but due to the centralized care model, outcome data were considered accurate and complete. In addition, we examined the association between 1-year mortality and four scoring systems—Japan SCORE, SPINACH SCORE, Clinical Frailty Scale (CFS), and Geriatric Nutritional Risk Index (GNRI)—to assess their utility as prognostic indicators. Furthermore, to evaluate the predictive accuracy of these scoring systems from short- to mid-term periods (up to 3 years), the area under the receiver operating characteristic (ROC) curve (AUC) was calculated. AUC values were obtained at 1-month intervals and then every 3 months postoperatively. In addition, time-dependent ROC analysis was performed to assess the temporal accuracy of each scoring system. These four scores were selected based on their complementary perspectives: the Japan SCORE is widely used in Japan as a surgical risk model for 30-day outcomes; the SPINACH SCORE was specifically developed to predict mid-term outcomes in CLTI patients; and the CFS quantifies biological frailty, whereas the GNRI reflects nutritional status—both of which are increasingly recognized as key factors in surgical risk stratification among older adults. Unlike the EuroSCORE II or STS score, these models also allow the integration of systemic vulnerabilities that may influence post-discharge outcomes in CLTI patients.

### 2.3. Therapeutic Strategy at Our Institute

A therapeutic strategy was determined for each patient based on the severity of cardiac disease and CLTI, the presence of other comorbidities, and the overall condition including frailty. When the cardiac condition was stable, priority was given to the treatment of CLTI. In contrast, for patients with unstable cardiac conditions, cardiac surgery was prioritized. However, in cases of severe limb ischemia, minimal lower extremity revascularization, such as bypass surgery under ultrasound-guided nerve block or endovascular therapy, was performed to avoid general anesthesia. Infected or necrotic tissue was debrided under local and/or regional anesthesia.

The choice between bypass surgery and endovascular therapy was based on general condition, mobility, and vein availability. Wound care and antibiotics were provided as needed, and treatment strategies were determined through a multidisciplinary heart–vascular team conference [5].

## 3. Scoring Systems

### 3.1. Japan SCORE

Japan SCORE is a risk prediction model developed collaboratively by the Japanese Society for Thoracic Surgery, the Japanese Association for Thoracic Surgery, and the Japanese Coronary Association to estimate perioperative risk in adult cardiac surgery [6,7,8]. Based on real-world data from Japanese patients, it is widely used to predict 30-day mortality and the incidence of major postoperative complications. The model incorporates a variety of preoperative variables, including age, sex, renal function, left ventricular ejection fraction (LVEF), urgency of the procedure, type of surgery, and comorbidities such as prior myocardial infarction or stroke. Japan SCORE plays a key role in clinical decision-making, risk stratification, and providing objective information during the informed consent process in the context of cardiac surgery in Japan.

### 3.2. SPINACH SCORE

In this study, we refer to the estimated survival rate derived from the SPINACH calculator as the “SPINACH SCORE” for convenience [9]. The SPINACH SCORE is based on the SPINACH study, a multicenter prospective registry in Japan, and incorporates multiple clinical variables, including age, dialysis dependence, history of heart disease, serum albumin level, WIfI classification, ambulatory status, and treatment modality (surgical or endovascular) [10]. It estimates 1- to 3-year overall survival in patients with CLTI. Given the high systemic risk burden in patients with CLTI, the SPINACH SCORE is a valuable tool for risk stratification and treatment decision-making in this critically ill population.

To examine the relationship between short- and mid-term prognoses and actual mortality, we used the estimated 30-day and 1-year survival values provided by the SPINACH SCORE. Although the SPINACH SCORE was originally developed to predict 1-year mortality in patients with CLTI, the original derivation study also reported 30-day mortality rates, and many of the included variables—such as renal replacement therapy, nutritional status, and mobility—are highly relevant to short-term surgical outcomes. In this study, we applied the SPINACH SCORE to evaluate 30-day mortality without recalibration to enable direct comparison with the Japan SCORE, which is specifically designed for perioperative risk prediction.

### 3.3. CFS

The Clinical Frailty Scale (CFS) is a widely used tool for assessing global frailty in older adults and assisting in the prediction of clinical outcomes and treatment decision-making [11]. Developed by Rockwood et al. in 2005 [11], the CFS classifies patients into nine categories, ranging from 1 (very fit) to 9 (terminally ill), based on overall physical functioning, comorbidities, and dependence in activities of daily living. It is a simple, rapid, and non-invasive assessment tool that relies on clinical judgment and patient history rather than laboratory or imaging data. The CFS has been validated as a prognostic indicator in various clinical contexts, including cardiac surgery and intensive care, where higher frailty scores are associated with an increased risk of postoperative complications and mortality [12].

### 3.4. GNRI

The Geriatric Nutritional Risk Index (GNRI) is a simple and objective tool developed to assess nutritional status and predict clinical outcomes in elderly patients. It is particularly relevant for older individuals with chronic illnesses or those undergoing surgery, as malnutrition in this population is associated with an increased risk of complications and mortality.

GNRI is calculated using the following formula:

GNRI = (14.89 × serum albumin [g/dL]) + (41.7 × current body weight/ideal body weight)

Ideal body weight is estimated based on height. Lower GNRI values indicate a higher nutritional risk. GNRI values are typically categorized as follows: <82 = major risk, 82–91.9 = moderate risk, 92–97.9 = mild risk, and ≥98 = no risk.

As a reproducible and quantitative index, GNRI has also been shown to correlate with postoperative outcomes in the field of cardiovascular surgery, making it a useful tool for preoperative risk stratification [13,14].

### 3.5. Statistical Analysis

All data analyses were performed using EZR version 4.2.2 (The R Foundation for Statistical Computing). Categorical variables were expressed as numbers (*n*) with percentages in parentheses and compared using the χ^2^ test. Continuous variables were presented as mean ± standard deviation or as median with interquartile range and were compared using the Mann–Whitney U test.

To determine the optimal cutoff values for the four scoring systems, receiver operating characteristic (ROC) curve analysis was conducted. Calibration was assessed using bootstrapped calibration plots with 100 repetitions. Decision curve analysis (DCA) was conducted using the rmda package in R, with 100 bootstrapped samples to estimate standardized net benefit across threshold probabilities from 0.01 to 0.50. Based on these cutoff values, patients were categorized into high-risk and low-risk groups, and survival probabilities were evaluated using Kaplan–Meier analysis.

Additionally, to assess the association between the scoring systems and mid-term prognosis, ROC curves were generated at one month postoperatively and subsequently every three months. The area under the curve (AUC) was calculated at each time point and visualized graphically. Furthermore, time-dependent ROC analysis was also conducted to account for the effect of survival time, and the corresponding AUCs were similarly plotted. A *p*-value of <0.05 was considered statistically significant.

## 4. Results

### 4.1. Baseline and Operative Characteristics

The breakdown of cardiac surgeries is presented in Table 1. Diabetes mellitus and end-stage renal disease requiring dialysis were common, both present in 36 patients (81.8%). The most frequently performed cardiac procedure was coronary artery bypass grafting, which was conducted in 35 cases (79.5%). This was followed by valve surgery, performed in 21 cases (47.7%), with some overlap between the procedures.

Comparisons were made between survivors and non-survivors at 30 days, as well as at 1 year postoperatively.

In the cohort of 44 patients, the 30-day mortality rate was 9.1%, and the 1-year mortality rate was 29.5%. The predicted 30-day mortality based on the Japan SCORE was 12.5%, while the 1-year predicted mortality according to the SPINACH SCORE was 18.5%, indicating a discrepancy between the predicted scores and the actual observed mortality rates (Table 2). In the comparison between 30-day survivors and non-survivors, significant differences were observed in left ventricular ejection fraction (LVEF) and postoperative hemodynamic instability. The survivor group had significantly higher LVEF and a lower incidence of preoperative hemodynamic instability than the non-survivor group (LVEF:49.2% vs. 23.5%, preoperative unstable hemodynamics: 5.0% vs. 75.0%, *p* < 0.05, Table 2). Furthermore, the foot infection grade was significantly lower in the survivor group than in the non-survivor group (0.0 vs. 1.5, *p*  <  0.05, Table 2). In addition, the 30-day predicted mortality calculated by the Japan SCORE was significantly lower in the survivor group (5.5% vs. 25.8%, *p*  <  0.05), and the rate of emergency operations was also significantly lower (20% vs. 75%, *p*  <  0.05, Table 2). Furthermore, in the comparison between 1-year survivors and non-survivors, a significant difference was observed only in the GNRI, with the survivor group showing a significantly higher GNRI (92.0 vs. 86.0, *p*  <  0.05, Table 2).

### 4.2. Association Between Four Scoring Systems and Short- and Mid-Term Mortality

#### 4.2.1. Japan Score

**Figure 2:**
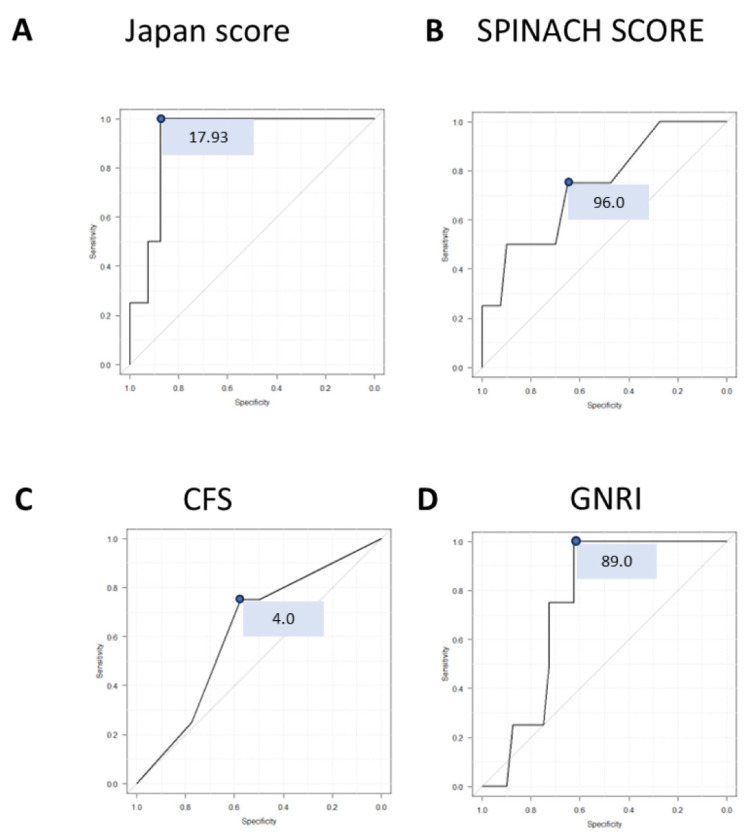
**ROC curve analysis for predictive accuracy identified cutoff values**. (**A**) For the Japan SCORE, the optimal cutoff value was determined based on the predicted 30-day mortality. ROC curve analysis identified 17.93 as the value with the highest combined sensitivity and specificity, and this was used as the cutoff. (**B**) For the SPINACH SCORE, the same method yielded a cutoff value of 96.0. (**C**) For the CFS, the calculated cutoff value was 4. (**D**) For the GNRI, the calculated cutoff value was 89.

Based on the receiver operating characteristic (ROC) curve analysis of the 30-day predicted mortality derived from the Japan score, a cutoff value of 17.93% was identified as optimal. Accordingly, patients were classified into a high-risk group (≥17.93%) and a low-risk group (<17.93%) (Figure 2A). According to the Japan score classification, significant differences were observed between the high-risk and low-risk groups in terms of LVEF and preoperative hemodynamic instability. The low-risk group had a significantly higher LVEF and a lower incidence of preoperative hemodynamic instability (LVEF: 50.6% vs. 36.0%, *p*  <  0.05; preoperative unstable hemodynamics: 0.0% vs. 33.3%, *p*  <  0.05; Supplementary Table S1).

#### 4.2.2. SPINACH SCORE

For the SPINACH SCORE, as with the Japan score, the 30-day predicted survival rate was calculated and analyzed. ROC curve analysis identified a cutoff value of 96.0, based on which patients were classified into high-risk and low-risk groups. Among the variables, only dialysis status showed a significant difference between the two groups, with the proportion of dialysis-dependent patients being significantly lower in the low-risk group (71.4% vs. 100%, *p*  <  0.05; Supplementary Table S2).

#### 4.2.3. CFS

For the CFS, ROC curve analysis identified a cutoff value of 4. Based on this threshold, patients were divided into two groups for further analysis; however, no significant differences were observed between the groups (Supplementary Table S3).

#### 4.2.4. GNRI

For the GNRI, patients were divided into two groups based on the cutoff value of 89.0 determined by ROC curve analysis. The prevalence of diabetes mellitus was significantly higher in the low-risk group (91.7% vs. 50.0%, *p*  <  0.05; Table S4), and serum albumin levels were also significantly higher in the low-risk group (3.3 vs. 2.3 g/dL, *p*  <  0.05; Supplementary Table S4).

### 4.3. Association Between Each Scoring System and Mid-Term Prognosis

**Figure 3:**
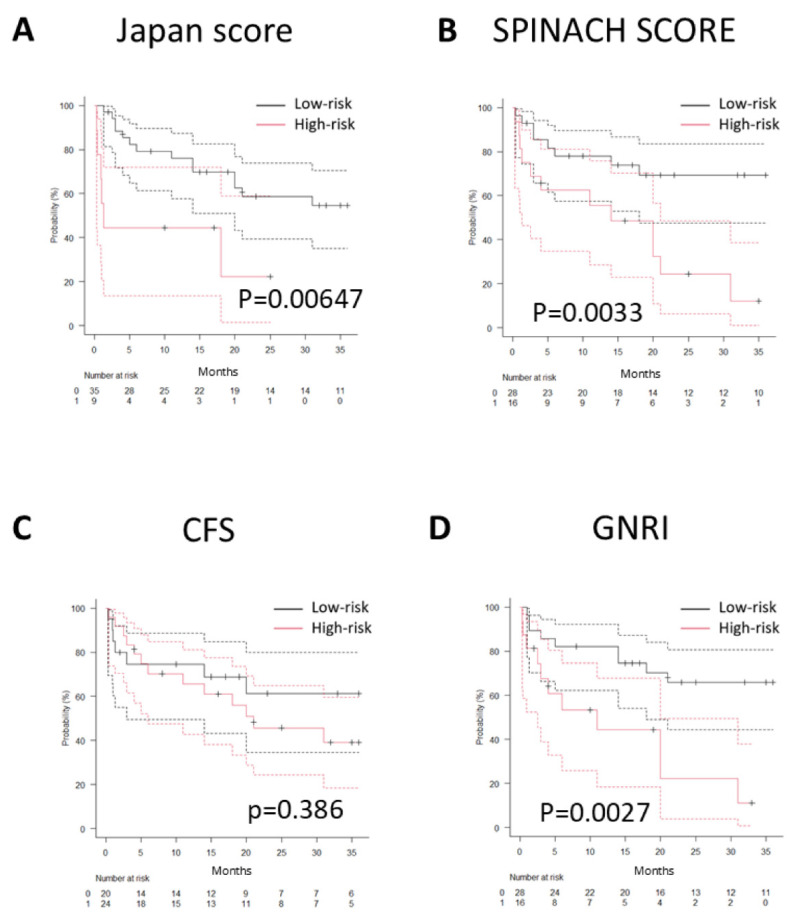
Association Between Each Scoring System and Mid-Term Prognosis. Kaplan–Meier survival curves according to risk stratification by four scoring systems. Dotted lines indicate 95% confidence intervals. (**A**) **Japan SCORE**: The low-risk group showed a significantly higher survival rate (*p*  <  0.05). (**B**) **SPINACH SCORE**: 30-day predicted survival values demonstrated significantly higher survival rates in the low-risk group (*p*  <  0.05). (**C**) **Clinical Frailty Scale (CFS)**: No significant difference in survival was observed between the high- and low-risk groups. (**D**) **Geriatric Nutritional Risk Index (GNRI)**: The low-risk group exhibited significantly higher survival (*p*  <  0.05).

**Figure 4:**
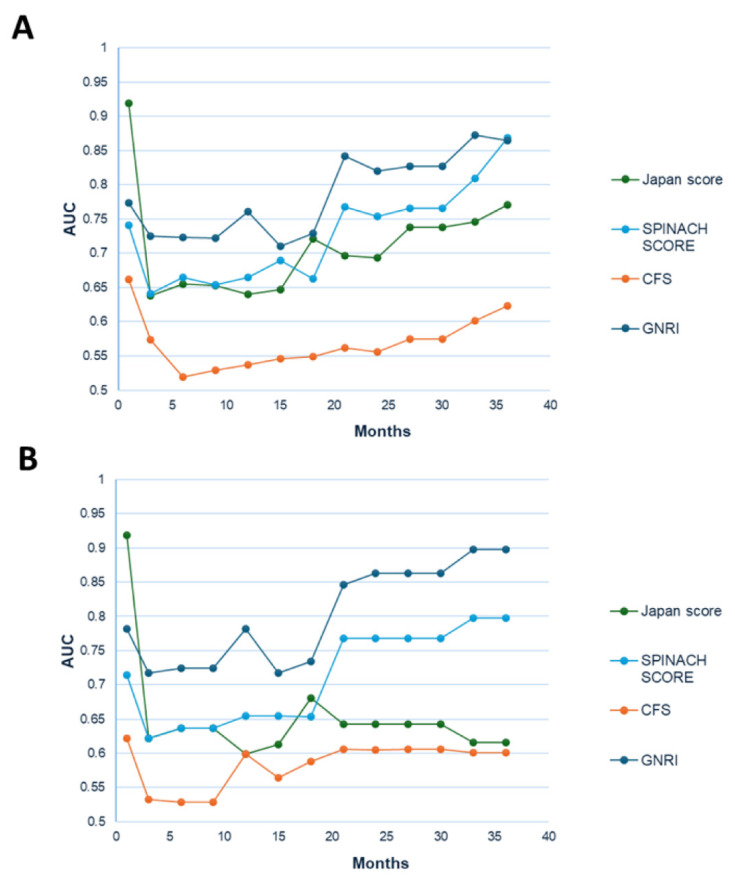
**Comparison of time-dependent changes in the area under the ROC curve (AUC)**. (**A**) From 1 month to 36 months postoperatively, the AUC values of ROC curves for each scoring system were calculated at 3-month intervals and plotted to generate a time series graph. The Japan SCORE showed the highest AUC at 1 month, but its values remained low thereafter. The CFS consistently demonstrated low AUC values throughout the entire observation period. In contrast, both the SPINACH SCORE and GNRI showed relatively high AUC values, particularly after 21 months. (**B**) To more accurately reflect survival duration in prognostic evaluation, time-dependent ROC curves were generated at corresponding intervals, and the AUC values at each time point were plotted. These time-dependent AUCs demonstrated similar trends.

The association between each scoring system (Japan SCORE, SPINACH SCORE, CFS, and GNRI) and mid-term prognosis (1-year survival) was analyzed to identify predictors of outcomes in patients with CLTI undergoing cardiac surgery. In the Kaplan–Meier analysis, the low-risk groups demonstrated significantly higher survival rates for the Japan SCORE, SPINACH SCORE (both 30-day and 1-year predictions), and GNRI. In contrast, no significant difference in prognosis was observed between the high- and low-risk groups for the CFS (Figure 3A–D). Furthermore, from 1 month to 36 months postoperatively, the AUC values of ROC curves for each scoring system were calculated at 3-month intervals and plotted to generate a time series graph (Figure 4A). The Japan SCORE showed the highest AUC at 1 month, but its values remained low thereafter. The CFS consistently demonstrated low AUC values throughout the entire observation period. In contrast, both the SPINACH SCORE and GNRI showed relatively high AUC values, particularly after 21 months. Additionally, to evaluate the ROC curves accounting for survival time, time-dependent ROC curves were generated at corresponding intervals, and the AUC values at each time point were plotted. These time-dependent AUCs showed similar trends (Figure 4B).

### 4.4. Calibration and Clinical Utility of Prognostic Models

Calibration plots demonstrated that both the Japan SCORE and SPINACH SCORE showed relatively good agreement between the predicted and observed probabilities. In contrast, the CFS and GNRI exhibited moderate miscalibration, particularly at higher predicted probability ranges.

DCA revealed that the GNRI and SPINACH SCORE offered greater net clinical benefit compared to strategies of treating all or none, especially across lower threshold probabilities (0.05–0.20). Conversely, the Japan SCORE and CFS showed limited clinical utility across most thresholds.

### 4.5. Additional Validation via Calibration and Decision Curve Analyses

To further evaluate the performance of the four prognostic models, we conducted calibration analyses using the rms package with 100 bootstrap resamples. All models demonstrated reasonable calibration, with bias-corrected calibration curves closely following the ideal 45-degree line. The mean absolute errors were 0.076 for the Japan SCORE, 0.028 for SPINACH SCORE, 0.047 for GNRI, and 0.047 for CFS.

We also performed DCA using the R rmda package. The net clinical benefit varied among the models depending on the threshold probability. Notably, the Japan SCORE and GNRI yielded the highest standardized net benefit across a clinically relevant range of thresholds (5–20%), supporting their potential utility in identifying high-risk patients who may benefit from aggressive perioperative management.

## 5. Discussion

There is currently no established treatment strategy for patients with CLTI who require cardiac surgery. For cardiovascular surgeons, the decision to proceed with high-risk cardiac surgery in patients with severely limited life expectancy due to CLTI remains a significant clinical dilemma. In this context, accurate prediction is essential to guide surgical decision-making. At our institution, the vascular surgery department is a high-volume center that actively performs both surgical and endovascular revascularization for lower extremity lesions, even in high-risk cases. Furthermore, recognizing that coronary artery disease is a major cause of mortality in patients with CLTI, we have adopted an aggressive approach to coronary revascularization with the goal of improving long-term survival. As a result, we have accumulated substantial clinical experience and focused our research efforts on this unique and high-risk population—patients undergoing cardiac surgery with comorbid CLTI [5].

Our findings suggest that the 30-day mortality predicted by the Japan SCORE may serve as a useful indicator of short-term prognosis. In contrast, both the GNRI and the predicted survival rates based on the SPINACH study may be more appropriate indicators for mid- to long-term outcomes (Figure 4). This sustained prognostic value of the SPINACH SCORE and GNRI beyond the early postoperative phase reflects the fact that long-term outcomes in patients with CLTI are likely influenced by factors related to systemic malnutrition, inflammation, and limb ischemia. Therefore, these indices may capture the broader burden of disease that persists after cardiac surgery. Incorporating these risk scores into preoperative assessments may allow for more comprehensive risk stratification, guiding decisions on surgical timing and the need for adjunctive care in vulnerable patients.

A comparison of the Japan SCORE and SPINACH calculator highlights a key difference in their evaluation of limb status. While the Japan SCORE includes only the presence of extracardiac vascular disease, the SPINACH calculator incorporates detailed limb assessments using the WIfI classification, as well as history of revascularization and amputation. These findings suggest that the severity of limb ischemia may influence long-term outcomes following cardiac surgery.

Previous studies have reported strong associations between undernutrition and poor limb- and life-related outcomes in CLTI patients [15,16,17,18]. In addition, preoperative nutritional status has been shown to correlate with mid- to long-term mortality following cardiac surgery, even in patients without CLTI [12]. Furthermore, a multicenter prospective study in Japan demonstrated that GNRI after revascularization independently predicted mortality, regardless of baseline GNRI [19]. These findings imply that appropriate nutritional management, even after surgery, may improve long-term outcomes.

In addition to malnutrition, frailty and sarcopenia have also been linked to poor prognosis in patients with LEAD [20,21]. In our study, we evaluated frailty using the CFS, focusing particularly on ambulatory status. However, no significant association with postoperative survival was observed. This may be attributed to limitations of the CFS in assessing walking ability in CLTI patients, whose mobility is often compromised by lower limb ischemia or activity restriction during treatment. A more nuanced evaluation of ambulation, possibly through structured interviews rather than visual assessment alone, may be required for accurate assessment.

Our findings highlight the challenges in prognosticating outcomes for CLTI patients undergoing cardiac surgery using conventional scoring systems. Although the Japan SCORE adequately predicts short-term surgical mortality, mid- to long-term outcomes may be more influenced by CLTI severity, frailty, and nutritional status, as reflected by the SPINACH SCORE and GNRI. Wakabayashi et al. reported that cardiac surgery in patients with advanced CLTI and high WIfI stages carries a particularly high risk [3]. Nevertheless, in cases with unstable hemodynamics, cardiac intervention cannot be delayed and must take precedence over limb revascularization. For patients identified as high risk through prognostic assessment, less invasive alternatives such as transcatheter aortic valve implantation or percutaneous coronary intervention may be considered depending on the clinical context. Therefore, when evaluating CLTI patients with severe cardiac disease, a comprehensive risk stratification approach that includes the Japan SCORE, SPINACH-based predicted survival, and GNRI may aid in tailoring treatment strategies and shared decision-making. However, further multicenter prospective studies are warranted to validate these findings and develop specific algorithms for this high-risk population.

## 6. Limitations

This study has several limitations. First, it was a retrospective, single-center analysis with a relatively small sample size, which may limit the generalizability of the findings. However, our institution is one of the few centers in Japan that actively performs both revascularization for CLTI and cardiac surgery, and all patients were managed and followed within a consistent institutional protocol. As a result, the dataset was complete with no missing values, and it reflects a highly specific and clinically relevant population.

Second, multivariable analysis was not performed. Both the Japan SCORE and SPINACH SCORE are composite indices derived from multivariable regression models; including these scores along with their individual components in the same model would likely introduce multicollinearity. Moreover, the small number of outcome events further limits the feasibility and reliability of multivariable modeling with multiple covariates.

Despite these limitations, our findings highlight the insufficient predictive performance of existing risk models in patients with CLTI undergoing cardiac surgery. This underlines the need for the development of novel, disease-specific risk stratification tools tailored to this high-risk population.

In future multicenter, prospective studies—especially in collaboration with other institutions that actively treat CLTI—validation of our findings and the construction of simplified clinical decision-making tools, such as treatment algorithms or nomograms, may become feasible and clinically impactful.

## 7. Conclusions

In this study, we evaluated prognostic indicators in CLTI patients undergoing cardiac surgery for severe heart disease. Our findings suggest that the Japan SCORE is useful for predicting short-term mortality, while SPINACH SCORE and GNRI are more relevant for mid- to long-term prognostication. Comprehensive assessment using these indicators from multiple perspectives is essential when considering surgical intervention in this high-risk patient population.

## Figures and Tables

**Table 1:** Baseline and operative characteristics of the patients.

Patients’ Characteristics	
Age (years, mean SD)	69.7 ± 9.9
Male (%)	29 (54.9%)
Diabetes mellitus (*n*, %)	36 (81.8%)
Hemodialysis (*n*, %)	36 (81.8%)
Serum albumin (g/dL, mean SD)	3.1 ± 0.5
Cardiac status	
LVEF (%, mean SD)	46.9 ±15.7
Coronary artery disease (*n*, %)	35
Severe aortic valve stenosis (*n*, %)	5.3 ± 1.7
Mitral valve stenosis (*n*, %)	2 (4.5)
Mitral valve regurgitation (*n*, %)	5 (11.4)
Infective endocarditis (*n*, %)	1 (2.3)
Preoperative unstable hemodynamics (*n*, %)	5 (11.4)
Operative characteristics	
coronary artery bypass grafting	35 (79.5%)
valve surgery	21 (47.7%)
others	10 (22.7%)

CFS: clinical frailty scale, GNRI: geriatric nutritional risk index, LVEF: left ventricular ejection fraction, SD; standard deviation.

**Table 2:** Comparison on patient characteristics, operative parameters and postoperative complications between 30 days/1-year survivors and non-survivors.

	Overall (*n* = 44)	30 Days Survivors and Non-Survivors			1 Year Survivors and Non-Survivors		
		Survivor (*n* = 40)	Non-Survivor (*n* = 4)	*p*-Value	Survivor (*n* = 31)	Non-Survivor (*n* = 13)	*p*-Value
Patient characteristics							
Age (median, IQR)	71.0 (63–78)	71 (62.5–76.5)	75.5 (68.3–81.5)	0.327	71 (62.0–86.0)	73.0 (65.0–78.0)	0.643
Male gender, *n* (%)	29 (65.9%)	26 (65.0)	3 (75.0)	NS	19 (61.3)	10 (76.9)	0.488
Diabetes mellitus, *n* (%)	36 (81.8%)	33 (82.5)	3 (75.0)	0.566	26 (83.9)	10 (76.9)	0.676
Insulin use, *n* (%)	11 (25.0%)	10 (25.0)	1 (25.0)	NS	8 (25.8)	3 (23.1)	NS
Hemodialysis, *n* (%)	36 (81.8%)	32 (80.0)	4 (100.0)	NS	23 (74.2)	13 (100.0)	0.082
Serum albumin, Median (g/dL, median, IQR)	3.2 (2.9–3.5)	3.2 (2.8–3.5)	3.1 (2.8–3.3)	0.728	3.2 (3.0–3.6)	2.9 (2.3–3.9)	0.107
C reactive protein (median, IQR)	1.65 (0.42–3.47)	2.19 (0.1–8.84)	1.50 (0.24, 3.26)	0.577	1.76 (0.1, 7.91)	3.00 (0.24, 8.84)	0.108
Cardiac status							
LVEF (median, IQR)	50.5 (34.8–60.90)	49 (22–68)	24 (16–31)	0.001 *	55.0 (25.0–60.0)	39.0 (28.0–57.0)	0.226
LVEF < 35%, *n* (%)	11 (25.0)	7 (17.5)	4 (100.0)	0.002 *	6 (19.4)	5 (38.5)	0.256
Coronary artery disease, *n* (%)	35 (79.5)	32 (80.0)	3 (75.0)	NS	25 (80.6)	10 (76.9)	NS
Acute coronary syndrome, *n* (%)	9 (20.5)	7 (17.5)	2 (50.0)	0.18	7 (22.6)	2 (15.4)	0.703
Aortic valve stenosis, *n* (%)	19 (43.2)	18 (45.0)	1 (25.0)	0.622	15 (48.4)	4 (30.8)	0.335
Mitral valve stenosis, *n* (%)	2 (4.5)	2 (5.0)	0 (0.0)	NS	2 (6.5)	0 (0.0)	NS
Mitral valve regurgitation, *n* (%)	5 (11.4)	5 (12.5)	0 (0.0)	NS	3 (9.7)	2 (15.4)	0.623
Infective endocarditis, *n* (%)	1 (2.3)	1 (2.5)	0 (0.0)	NS	0 (0.0)	1 (7.7)	0.295
Preoperative unstable hemodynamics, *n* (%)	5 (11.4)	2 (5.0)	3 (75.0)	0.003	2 (6.5)	3 (23.1)	0.144
Limb status; the three major factors of WIfi							
Wound grade (median, IQR)	2.0 (1.0–2.0)	2.0 (1.0–2.0)	2.0 (1.5–2.3)	0.546	2.0 (1.0–2.0)	2.0 (1.0–2.0)	0.415
Ischemic grade (median, IQR)	3.0 (2.0–3.0)	3.0 (2.0–3.0)	3.0 (2.0–2.3)	0.119	2.0 (2.0–3.0)	2.0 (2.0–3.0)	0.519
Foot infection grade (median, IQR)	3.0 (2.0–3.0)	**0 (0.0–1.0)**	**1.5 (0.8–2.3)**	0.041 *	0.0 (0.0–1.0)	0.0 (0.0–1.0)	0.917
Scores of 4 different systems							
Japan SCORE (median, IQR)	12.45 (14.74)	**5.5 (3.2–13.5)**	**25.8 (18.1–25.8)**	0.000663 *	5.51 (3.0–13.7)	7.79 (3.5–18.2)	0.252
SPINACH SCORE (median, IQR) **	81.5 (67.0–89.3)	82.0 (68.8–90.0)	66.0 (51.5–66.0)	0.111	84.0 (72.0–90.5)	75.0 (61.0–85.0)	0.082
CFS (median, IQR)	5.5 (4.0–7.0)	6.5 (4.0–7.0)	4.0 (3.8–4.8)	0.405	5.0 (3.5–7.0)	7.0 (4.0–7.0)	0.508
GNRI (median, IQR)	91.0 (85.5–97.8)	91.25 (85.5–98.2)	86.0 (84.5–87.0)	0.116	**92.0 (87.7–121.0)**	**86.0 (85.1–99.8)**	0.0108 *
Surgical procedures							
Operation time (median, IQR)	320.5 (257.5–369.5)	309.5 (257.5–396.5)	351.5 (294.5–396.5)	0.791	320.0 (246.5–399.0)	324.0 (264.0–366.0)	0.908
CPB time (median, IQR)	134.0 (71.8–176.0)	129.5 (70.8–172.3)	189.5 (125.8–246.3)	0.219	135.0 (78.5–173.5)	123.0 (62.0–213.0)	0.907
Aortic clamp time (median, IQR)	71.0 (0.0–123.3)	70.5 (0–119.5)	97.0 (64.8–134.5)	0.449	82.0 (0.0–121.0)	49.0 (0.0–354.0)	0.618
Emergency operation, *n* (%)	**11 (25.0)**	**8 (20.0)**	**3 (75.0)**	0.043 *	8 (25.8)	3 (23.1)	NS
Coronary artery bypass grafting, *n* (%)	35 (79.5)	32 (80.0)	3 (75.0)	NS	25 (80.6)	10 (76.9)	NS
Off-pump CABG, *n* (%)	8 (18.2)	8 (20.0)	0 (0.0)	NS	6 (19.4)	2 (15.4)	NS
Aortic valve replacement, *n* (%)	17 (38.6)	16 (40.0)	1 (25.0)	NS	14 (45.2)	3 (23.1)	0.198
Mitral valve repair/replacement, *n* (%)	5 (11.4)	5 (12.5)	0 (0.0)	NS	3 (9.7)	2 (15.4)	0.623
Postoperative complications							
Severe infections, *n* (%)	4 (9.1)	3 (7.5)	1 (25.0)	0.327	1 (3.2)	3 (23.1)	0.071
Mediastinitis, *n* (%)	7 (15.9)	7 (17.5)	0 (0.0)	NS	5 (16.1)	2 (15.4)	NS
Heart failure, *n* (%)	4 (9.1)	2 (5.0)	2 (50.0)	0.036	1 (3.2)	3 (23.1)	0.071
Sudden cardiopulmonary arrest, *n* (%)	5 (11.4)	3 (7.5)	2 (50.0)	0.057	1 (3.2)	4 (30.8)	0.022
Cerebral infarction, *n* (%)	2 (4.5)	2 (5.0)	0 (0.0)	NS	1 (3.2)	1 (7.7)	0.508
Non-occlusive mesenteric ischemia, *n* (%)	1 (2.3)	0 (0.0)	1 (25.0)	0.091	0 (0.0)	1 (7.7)	0.295

* *p* value < 0.05; ** As the SPINACH score includes predicted mortality at both 30 days and 1 year, we applied it separately to each time point. CFS: clinical frailty scale, CPB: cardiopulmonary bypass, CABG: coronary artery bypass grafting, GNRI: geriatric nutritional risk index, IQR: interquartile range, LVEF: left ventricular ejection fraction; NS: not significant.

## Data Availability

The raw data supporting the conclusions of this article will be made available by the authors on request.

## Supplementary Materials

The following supporting information can be downloaded at: https://www.mdpi.com/article/10.3390/jcm14176210/s1, Table S1: Comparison between high risk and low-risk groups according to the 30-day predicted mortality estimated by the Japan score; Table S2: Comparison between high-risk and low-risk group based on the 30-day and 1-year predicted survival rate from the SPINACH SCORE; Table S3: Comparison between high-risk and low-risk group based on the CFS; Table S4: Comparison between high-risk and low-risk group based on the GNRI.

## Abbreviations

AUC	Area Under the Curve
CFS	Clinical Frailty Scale
CLTI	Chronic Limb-Threatening Ischemia
GNRI	Geriatric Nutritional Risk Index
LEAD	Lower Extremity Arterial Disease
LVEF	Left Ventricular Ejection Fraction
ROC	Receiver Operating Characteristic
WIfI	Wound, Ischemia, and foot Infection
