# Supplementary material for: Mid-Term Mortality Prediction Using Four Established Risk Scores in Patients with Chronic Limb-Threatening Ischemia Undergoing Cardiac Surgery
Source: J Clin Med. 2025 Sep 2;14(17):6210. doi: 10.3390/jcm14176210 (PMC12429257; doi:10.3390/jcm14176210)
Supplement: Supplementary file 1 [file jcm-14-06210-s001.zip › jcm-3844694-supplementary.pdf]

**Table S1.** Comparison between high risk and low-risk groups according to the 30-day predicted mortality estimated by the Japan score.

| | **Low-risk group (N=35)** | **High-risk group (N=9)** | **p value** |
|---|---|---|---|
| Patient's characteristics | | | |
| Age (median, IQR) | 70.00 (9.82) | 68.67 (10.64) | 0.723 |
| Male gender, n (%) | 22 ( 62.9) | 7 ( 77.8) | 0.695 |
| Diabetes mellitus, n (%) | 30 ( 85.7) | 7 ( 77.8) | 0.619 |
| Hemodialysis, n (%) | 27 ( 77.1) | 9 (100.0) | 0.175 |
| Serum albumin (g/dL, median, IQR)) | 3.13 (0.58) | 3.07 (0.34) | 0.75 |
| C reaction protein (mg/dL, median, IQR) | 2.08 (2.40) | 2.32 (2.08) | 0.781 |
| Cardiac status | | | |
| LVEF (%, median, IQR) | 50.6(14-30) | 36.0 (16-60) | 0.011* |
| Coronary artery disease , n (%) | 27 ( 77.1) | 8 ( 88.9) | 0.659 |
| Severe aortic valve stenosis, n (%) | 15 ( 42.9) | 4 ( 44.4) | NS |
| Mitral valve stenosis, n (%) | 1 ( 2.9) | 0 ( 0.0) | NS |
| Mitral valve regurgitation, n (%) | 5 ( 14.3) | 0 ( 0.0) | 0.566 |
| Infective endocarditis, n (%) | 1 ( 2.9) | 0 ( 0.0) | NS |
| Preoperative unstable hemodynamics, n (%) | 0 ( 0.0) | 3 ( 33.3) | 0.006 |

*P value<0.05; IQR: interquartile range, LVEF: left ventricular ejection fraction; NS: not significant.

**Table S2.** Comparison between high-risk and low-risk group based on the 30-day and 1-year predicted survival rate from the SPINACH SCORE.

| | **Low -risk group (N=28)** | **High risk group (N=16)** | **p value** |
|---|---|---|---|
| Patient's characteristics | | | |
| Age (median, IQR) | 69.0 (59.0-76.5) | 73.5 (70.6-78.3) | 0.0967 |
| Male gender, n (%) | 19 (67.9) | 10 (62.5) | 0.751 |
| Diabetes mellitus, n (%) | 23 (82.1) | 13 (81.3) | NS |
| Hemodialysis, n (%) | 20 (71.4) | 16 (100.0) | 0.0365* |
| Serum albumin (g/dL, median, IQR)) | 3.2 (3.1-3.6) | 3.0 (2.6-3.4) | 0.126 |
| C reaction protein (mg/dL, median, IQR) | 1.10 (0.23-2.76) | 1.22 (0.58-4.54) | 0.246 |
| Cardiac status | | | |
| LVEF (%, median, IQR) | 52.0 (35.0-60.0) | 44.5 (26.5-60.0) | 0.406 |
| Coronary artery disease , n (%) | 23 (82.0) | 12 (75.0) | 0.702 |
| Severe aortic valve stenosis, n (%) | 11 (39.3) | 8 (50.0) | 0.54 |
| Mitral valve stenosis, n (%) | 2 (7.1) | 0 (0.0) | 0.526 |
| Mitral valve regurgitation, n (%) | 4 (14.3) | 1 (6.3) | 0.638 |
| Infective endocarditis, n (%) | 0 (0.0) | 1 (6.3) | 0.364 |
| Preoperative unstable hemodynamics, n (%) | 3 (10.7) | 2 (12.5) | NS |

*P value<0.05; IQR: interquartile range, LVEF: left ventricular ejection fraction; NS: not significant.

**Table S3.** Comparison between high-risk and low-risk group based on the CFS.

| Variable | Low-risk group (N=20) | High-risk group (N=24) | p value |
|---|---|---|---|
| Patient's characteristics | | | |
| Age (median, IQR) | 73.5 (67.3-79.0) | 70.0 (73.0-75.8) | 0.238 |
| Male gender, n (%) | 16 (80.0) | 13 (54.2) | 0.111 |
| Diabetes mellitus, n (%) | 15 (75.0) | 21 (87.5) | 0.436 |
| Hemodialysis, n (%) | 14 (70.0) | 22 (88.0) | 0.115 |
| Serum albumin, median (g/dL) | 3.3 (3.1-3.6) | 3.1 (2.5-3.4) | 0.068 |
| C reaction protein(mg/dL, median, IQR) | 0.55 (0.15-2.33) | 1.44 (0.82-4.06) | 0.039 |
| Cardiac status | | | |
| LVEF (%, median, IQR) | 50.5 (32.5-50.0) | 52.5 (35.0-60.0) | 0.75 |
| Coronary artery disease , n (%) | 17 (85.0) | 18 (75.0) | 0.477 |
| Severe aortic valve stenosis, n (%) | 7 (35.0) | 12 (50.0) | 0.361 |
| Mitral valve stenosis, n (%) | 1 ( 5.0) | 1 (4.2) | NS |
| Mitral valve regurgitation, n (%) | 0 ( 0.0) | 5 (20.8) | 0.053 |
| Infective endocarditis, n (%) | 0 ( 0.0) | 1 (4.2) | 0.477 |
| Preoperative unstable hemodynamics, n (%) | 3 (15.0) | 2 (8.3) | 0.646 |

*P value<0.05; CFS: clinical frailty scale, IQR: interquartile range, LVEF: left ventricular ejection fraction; NS: not significant.

**Table S4.** Comparison between high-risk and low-risk group based on the GNRI.

| | Low-risk group (N=28) | High-risk group (N=16) | p value |
|---|---|---|---|
| Patient's characteristics | | | |
| Age (median, IQR) | 69.0 (59.0-75.3) | 74.5 (71.5-78.0) | 0.052 |
| Male gender, n (%) | 19 ( 69.4) | 10 ( 50.0) | 0.414 |
| Diabetes mellitus, n (%) | 26 ( 91.7) | 10 ( 50.0) | 0.014 |
| Hemodialysis, n (%) | 21 ( 80.6) | 15 ( 87.5) | NS |
| Serum albumin, median (g/dL) | 3.3 (3.1-3.6) | 2.3 (2.4-3.1) | 0.002* |
| C reaction protein(mg/dL, median, IQR) | 1.16 (0.24-3.45) | 1.19(0.51-2.93) | 0.6 |
| Cardiac status | | | |
| LVEF (%, median, IQR) | 50.0 (32.3-59.3) | 57.0 (38.0-60.5) | 0.386 |
| Coronary artery disease , n (%) | 24 ( 83.3) | 11 ( 62.5) | 0.329 |
| Severe aortic valve stenosis, n (%) | 13 ( 47.2) | 6 ( 25.0) | 0.433 |
| Mitral valve stenosis, n (%) | 1 ( 2.8) | 1 ( 0.0) | NS |
| Mitral valve regurgitation, n (%) | 3 ( 11.1) | 2 ( 12.5) | NS |
| Infective endocarditis, n (%) | 0 ( 0.0) | 1 ( 12.5) | 0.182 |
| Preoperative unstable hemodynamics, n (%) | 2 ( 5.6) | 3 ( 12.5) | 0.461 |

*P value<0.05; GNRI: geriatric nutritional risk index, IQR: interquartile range, LVEF: left ventricular ejection fraction; NS: not significant.
